# Synthesis and Biological Evaluation of Novel *N*-Methyl-picolinamide-4-thiol Derivatives as Potential Antitumor Agents

**DOI:** 10.3390/molecules17066317

**Published:** 2012-05-25

**Authors:** Ting-Ting Huang, Yun-Chuang Huang, Xiao- Yu Qing, Yong Xia, Xun Luo, Ting-Hong Ye, Luo-Ting Yu

**Affiliations:** State Key Laboratory of Biotherapy, West China Hospital, West China Medical School, Sichuan University, Chengdu 610041, China; Email: huangtingting5400@qq.com (T.-T.H.); hyc159@gmail.com (Y.-C.H.); qingxiaoyu5126@163.com (X.-Y.Q.); zhangweihuascu@163.com (Y.X.); luoxun_cn@126.com (X.L.); yetinghong0908@163.com (T.-H.Y.)

**Keywords:** *N*-methylpicolinamide-4-thiol derivatives, antitumor, Aurora-B kinase, docking study

## Abstract

A novel series of *N*-methylpicolinamide-4-thiol derivatives were synthesized and evaluated on human cancer cell lines. Among them, compound **6p** displayed potent and broad-spectrum anti-proliferative activities *in vitro* on some human cancer cell lines, even better than sorafenib. The advanced kinase inhibitory assays showed that compound **6p** could selectively inhibit Aurora-B kinase. The biological results were rationalized by the molecular docking study, which indicated the stable interactions of **6p** with the Aurora-B kinase.

## 1. Introduction

Aurora proteins-A, -B, and -C, a small family of serine/threonine kinases [1], play distinct roles in the regulation of mitosis [2]. Aurora-A and -B are known to be frequently overexpressed in a wide range of different human tumors, including breast, colon, lung, ovarian, and pancreatic cancers [3,4,5,6], suggesting their potential role in tumorigenesis [7].

**Figure 1:**
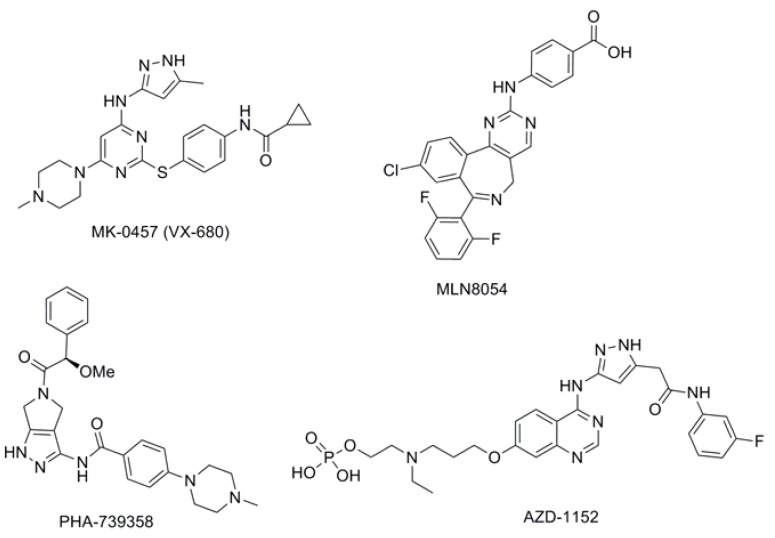
Inhibitors of Aurora kinases.

In recent years, the Aurora proteins have been actively pursued as anticancer targets for the discovery of new cancer chemotherapeutics. As a result, several small-molecule inhibitors of the Aurora kinases have been identified, some of which have reached clinical evaluation, including MK-0457(VX-680) [8,9], MLN8054 [10], PHA-739358 [11] and AZD-1152 [12] (Figure 1). However, the ideal inhibitor profile for therapeutic use is still unclear, and these inhibitors with complex structures are difficult to synthesize.

**Figure 2:**
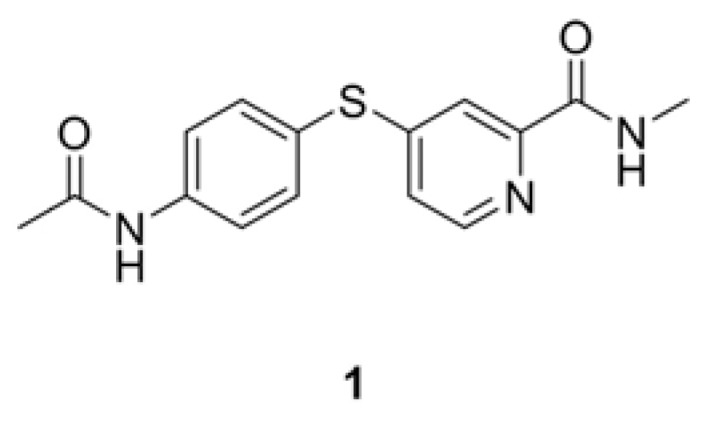
Structure of compound **1**.

Our group has been interested in the design, screening, synthesis and biological evaluation of novel tumor growth inhibitors. In a previous cell-based screening of our privileged small molecule library, we found that a drug-like compound, *N*-methylpicolinamide-4-thiol (Figure 2), exhibited moderate *in* *vitro* cytotoxicity against human hepatocellular carcinoma cell line HepG2 (IC_50_ = 62.96 µM). In order to find more potent antiproliferative compounds, we designed and synthesized a series of novel *N*-methylpicolinamide-4-thiol derivatives based on compound **1**, employing the structure-activity relationship (SAR) study.

## 2. Results and Discussion

### 2.1. Chemistry

**Scheme 1:**
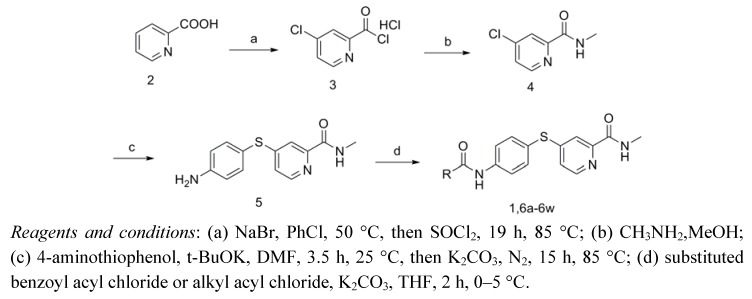
Synthetic route to compounds **1** and **6a–w**.

The synthetic route for the target compounds **1** and **6a–w** is shown in Scheme 1. Compound **3** was synthesized according to the reported method with a small change [13]. Treatment of 2-picolinic acid (**2**) with SOCl_2_ in the presence of NaBr and chlorobenzene afforded acid chloride **3** as the corresponding HCl salt. This HCl salt was then treated with methylamine solution (2.0 mol/L) in methanol to yield **4**. To obtain **5** [14], compound **4** and potassium carbonate were treated with a solution of 4-aminothiophenol, which had been stirred at room temperature for 3.5 h in the presence of potassium tert-butoxide in dry *N,N*-dimethylformamide. The contents were then heated to 85 °C under argon for 15 h. Acylation of the amino group of **5** with different substituted benzoyl chlorides or alkyl acyl chlorides yielded the target compounds **1** and **6a–w** [15]. The structures of compounds **1** and **6a–w** were fully characterized by ^1^H-NMR, ^13^C-NMR and ESI-MS analysis.

### 2.2. Biological Evaluation

**Figure 3:**
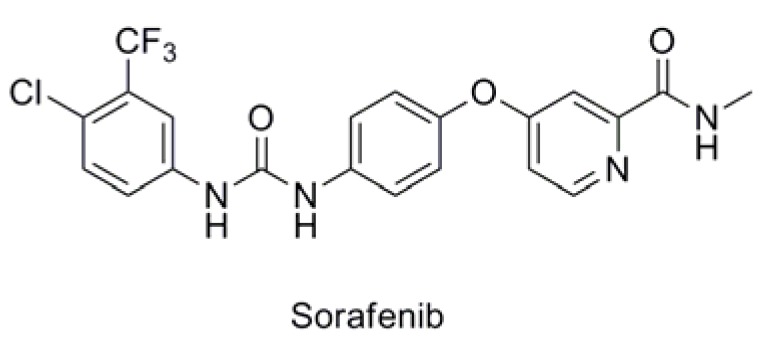
Structure of Sorafenib.

As shown in Table 1, twenty-three *N*-methylpicolinamide-4-thiol derivatives were synthesized to survey the SAR by evaluating the cell growth inhibitory activity in human liver hepatocellular carcinoma (HepG2) cells. Sorafenib (Figure 3), which was found to significantly prolong the survival of advanced hepatocellular carcinoma (HCC) patients, was selected as the positive control.

Just as seen in the phenyl series **6a–o**, unsubstituted phenyl analog **6a** showed good inhibitory activity, IC_50_ = 16.54 μM. As for the methoxy group, the activity of the analogue with methoxyl at*meta-*position (**6b**, IC_50_ = 15.43 μM) is better than that of the *para-*(**6c**) and *ortho*-(**6d**, IC_50_ = 23.91 μM) substituted analogues. Compound **6e (**IC_50_ = 7.12 μM**)**, with two methoxy groups substituted at the *meta-*position, was one of the most potent inhibitors in this series. In terms of halogen atoms (compounds **6f–j**), the location had little effect on the activities. However, when the number of the halogen atoms was two, the activity of **6h** (IC_50_ = 10.55 μM) and **6i** improved greatly. Introduction of the electron-withdrawing groups CF3 [**6k** (IC_50_ = 17.04 μM), **6l** (IC_50_ = 10.96 μM), **6m**] and NO2 [**6n** (IC_50_ = 19.12 μM), **6o**] on the phenyl ring at the *meta*- and *para*-position was tolerated.

Replacement of the benzyl group with an aliphatic group was also tolerated. With the IC_50_ value of 2.23 μM, compound **6p**, which exhibited a nearly 15-fold improvement in inhibitory activity against HepG2 cells over that of sorafenib (IC_50_ = 16.30 μM), was the most potent analogue in this series.

The number of chloride atom and the length of the carbon chain had a great effect on the activities. The activity was lost when the carbon chain was extended to chloropropyl or butyl chloride structures (compounds **6s,t**). The introduction of two or three chloride atoms (compounds **6q**,**r**) also had a detrimental effect on the potency. In addition, unsubstituted aliphatic derivatives **6u**–**w** were also tested, but their activities were not notable.

**Table 1:** Inhibition of cell proliferation by compounds **6a–w**. 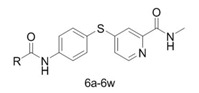

Compound	R	IC_50_(µM) ^a^	Structure of the compound
		HepG2	
**6a**	-phenyl	16.54	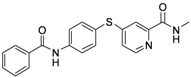
**6b**	-phenyl-*m*-OCH_3_	15.43	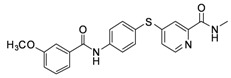
**6c**	-phenyl-*p*-OCH_3_	46.97	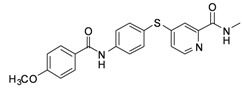
**6d**	-phenyl-*o*-OCH_3_	23.91	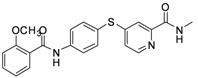
**6e**	-phenyl-3,5-Di-OCH_3_	7.12	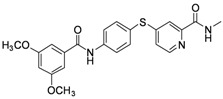
**6f**	-phenyl-*o*-Cl	61.55	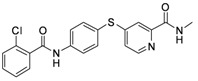
**6g**	-phenyl-*m*-F	48.93	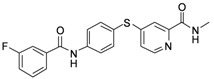
**6h**	-phenyl-2,4-Di-Cl	10.55	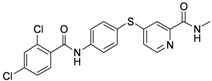
**6i**	-phenyl-2,6-Di-F	23.25	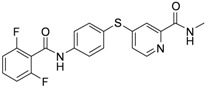
**6j**	-phenyl-2,3,4,5-Tetra-F	54.24	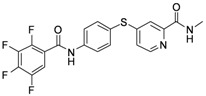
**6k**	-phenyl-*m*-CF_3_	17.04	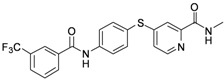
**6l**	-phenyl-*p*-CF_3_	10.96	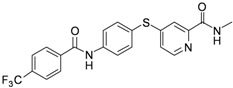
**6m**	-phenyl-*o*-CF_3_	65.38	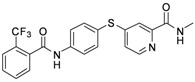
**6n**	-phenyl-*m*-NO_2_	19.12	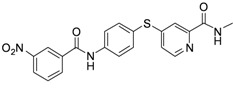
**6o**	-phenyl-*p*- NO_2_	33.67	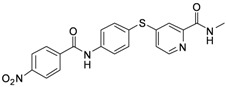
**6p**	-CH_2_Cl	2.23	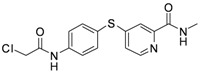
**6q**	-CHCl_2_	44.09	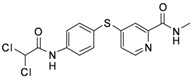
**6r**	-CCl_3_	94.55	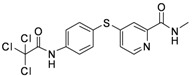
**6s**	-CH_2_CH_2_Cl	70.09	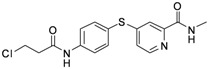
**6t**	-CH_2_CH_2_CH_2_Cl	81.53	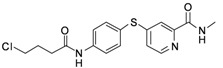
**6u**	-CH_2_CH_3_	75.54	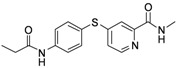
**6v**	-CH_2_CH_2_CH_3_	180.31	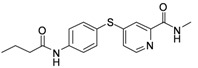
**6w**	-C(CH_3_)_3_	41.15	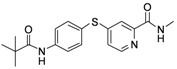
**Sorafenib**		16.30	

^a^ Values are means of three independent experiments.

To further study the cytotoxic profile, the most potent compound **6p** was selected for further evaluation of its inhibitory activity against a panel of human cancer cell lines. Interestingly, compound **6p** showed broad-spectrum antiproliferative activities *in vitro* (Table 2). It had significant cytotoxicity against colon cancer cell lines HCT-116 and SW480, lung cancer cell line SPC-A1 and melanotic cancer cell line A375 with IC_50_ values <10 μM.

**Table 2:** Inhibition of cell proliferation by compound **6p** and Sorafenib.

Compound	IC_50_ (µM) ^a^							
	HepG2	MCF-7	HCT116	SW480	A549	SPC-A1	A375	U87
**6p**	2.23	35.73	9.14	8.78	13.71	9.61	6.97	25.53
Sorafenib	16.30	>100	10.09	40.65	13.15	18.60	17.96	62.19

^a^ Values are means of three independent experiments.

Compound **6p** was evaluated on six kinases at a concentration of 10 μM (Table 3). It was found that **6p** could selectively inhibit Aurora-B kinase to a significant level (87% inhibition at 10 μM). This result provided a possible reason for its broad-spectrum antiproliferative activities.

**Table 3:** Kinase inhibitory assays of **6p**.

Compound	% inhibition at 10 μM					
	Aurora-A	Aurora-B	Axl	Flt3	KDR	PDGFRα
**6p**	−18	87	−1	−6	7	0

### 2.3. Molecular Docking Study

In order to further investigate the interactions between compound **6p** and the Aurora-B kinase, a docking study was performed using the Genetic Optimization for Ligand Docking (GOLD) 4.0 program. The crystal structure of Aurora-B (PDB Code: 4AF3) was used as the reference receptor.

**Figure 4:**
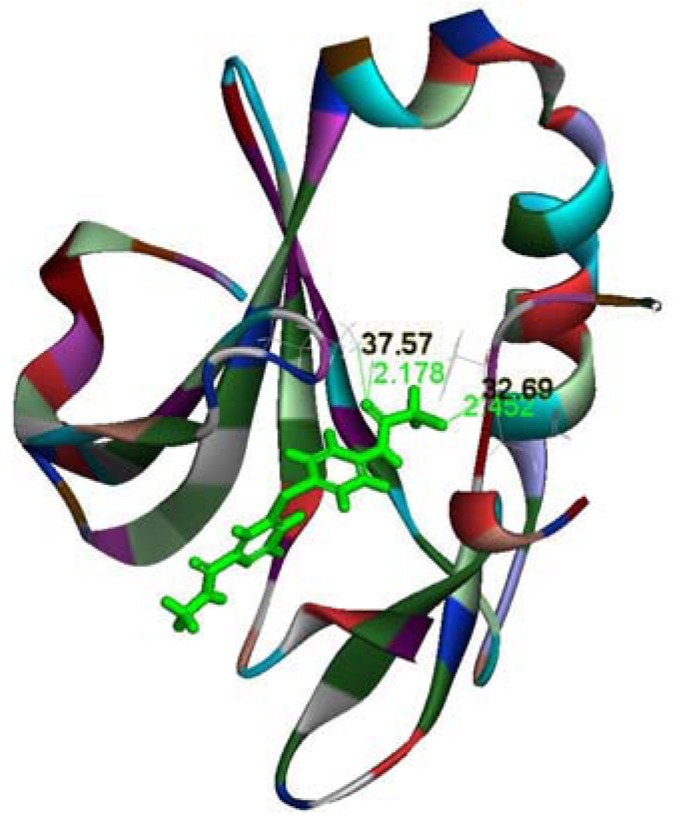
The binding mode of compound **6p** with ATP pocket of Aurora-B kinase obtained by molecular docking experiments (PDB code: 4AF3).

According to the docking result, we can discern that two hydrogen bonds were formed between compound **6p** and the Aurora-B kinase. As shown in Figure 4, the chlorine of the inhibitor formed one hydrogen bond with the residue Phe219 (2.452 Å, 32.69°), and another hydrogen bond was found between the residue Lys106 and the carbonyl group near the chlorine of the inhibitor (2.178 Å, 37.57°). In addition, there was a π-π conjugation interaction between the benzene ring group of compound **6p** and the Phe88 residue in the binding mode. The stable interactions between the inhibitor and Aurora-B kinase rationalize the obtained biological results.

## 3. Experimental

### 3.1. General

The human cancer cell lines were purchased from the American Type Culture Collection (ATCC, Rockville, MD, USA). Dulbecco’s modified Eagle medium (DMEM) and RPMI 1640 were purchased from Gibco (Grand Island, NY, USA). Fetal bovine serum (FBS) was purchased from Hyclone (Logan, UT, USA). Melting points were determined on a SGW X-4 microscopic melting point (Shanghai Precision & Scientific Instrument Co., Ltd, Shanghai, China). ^1^H-NMR and ^13^C-NMR spectra were recorded on a Bruker Varian Unity Inova-400 (400/100 MHz) spectrometer using TMS as internal reference chemical. Shifts are expressed as δ values in ppm. Mass spectra (MS) were measured on a Q-TOF Premier mass spectrometer (Micromass, Manchester, UK) utilizing electrospray ionization (ESI).

### 3.2. Preparation of 4-Chloropyridine-2-carbonyl Chloride Hydrochloride *(**4**)*

4-Chloropyridine-2-carbonyl chloride hydrochloride was prepared according to the reported method. Thionyl chloride (198 g, 1.68 mol) was added to a mixture of 2-picolinic acid (60 g, 0.48 mol), sodium bromide (8.08 g, 0.0785 mol) and chlorobenzene (84.8 g). Then 2.0 M methylamine solution in methanol was added to afford **4** as a white solid. Yield: 48 g, 58%; m.p. 34.0–38.0 °C; ^1^H-NMR (DMSO-*d*_6_): δ 3.04 (d, *J* = 5.2 Hz, 3H), 7.43 (dd, *J* = 5.2, 2.0 Hz, 1H), 7.98 (s, 1H), 8.21 (d, *J* = 1.6 Hz, 1H), 8.44 (d, *J* = 5.2 Hz, 1H); ESI-MS: *m/z* 193.04 (M+Na)^+^.

### 3.3. Preparation of 4-(4-Aminophenylthio)-N-methylcarboxamide *(**5**)*

Potassium tert-butoxide (4.04 g, 36.00 mmol) was added to a stirred solution of 4-aminothiophenol (3.76 g, 30.00 mmol) in dry *N*,*N*-dimethylformamide (58.68 mL), and the reddish-brown mixture was stirred at room temperature for 3.5 h. To the mixture was added **4** (5.89 g, 34.50 mmol) and potassium carbonate (25.44 g, 180.00 mmol), and then stirred at 85 °C under nitrogen for 15 h. The mixture was cooled to room temperature and poured into the mixture of ethyl acetate (200 mL) and brine (200 mL). The aqueous layer was extracted with ethyl acetate (150 mL). The combined organic layers were washed with brine (3 × 400 mL), dried over sodium sulfate, and concentrated to afford **5** as an orange solid. Yield: 6.76 g, 87%; m.p. 112.8–115.1 °C; ^1^H-NMR (DMSO-*d*_6_): δ 2.81 (q, *J* = 10.0 Hz, 3H), 5.74 (s, 2H), 6.68 (d, *J* = 8.4 Hz, 2H), 7.18 (dd, *J* = 3.6, 23.6 Hz, 3H), 7.49 (s, 1H), 8.34 (d, *J* = 5.2 Hz, 1H), 8.71 (d, *J* = 4.4 Hz, 1H); ^13^C-NMR (DMSO*-d*_6_): δ 164.43, 154.72, 151.47, 150.44, 148.39, 137.46 (2C), 121.94, 117.37, 115.57 (2C), 110.60, 26.42; ESI-MS: *m/z* 282.29 (M+Na)^+^.

### 3.4. General Procedure for Preparing Compounds ***1*** and ***6a–w***

Compound **5** (0.52 g, 2.00 mmol) and anhydrous potassium carbonate (0.69 g, 5.00 mmol) were suspended in THF (7.00 mL), and then different substituted benzoyl chlorides or alkyl acyl chlorides (2.10 mmol) was added dropwise at 0–5 °C. The mixture was stirred at room temperature for 2 h and poured into the mixture of ethyl acetate (50 mL) and brine (50 mL). The aqueous layer was back extracted with ethyl acetate (3 × 30 mL). The combined organic layers were washed with brine (3 × 50 mL), dried over sodium sulfate, and concentrated to afford compounds **6a–w**.

*4-(4-Benzamidophenylthio)-N-methylpicolinamide* (**6a**). Orange solid; yield: 91.87%; m.p. 139.1–140.7 °C; ^1^H-NMR (DMSO-*d*_6_): δ 2.77 (d, *J* = 4.4 Hz, 3H), 7.27 (dd, *J* = 2.0, 5.2 Hz, 1H), 7.56 (t, *J* = 7.4 Hz, 2H), 7.61–7.64 (m, 4H), 8.02 (q, *J* = 7.86 Hz, 4H), 8.42 (d, *J* = 5.2 Hz, 1H), 8.84 (d, *J* = 4.4 Hz, 1H), 10.63 (s, 1H); ^13^C-NMR (DMSO*-d*_6_): δ 166.48, 164.30, 152.61, 150.64, 148.71, 141.69, 136.56 (2C), 135.19, 132.27, 128.91 (2C), 128.25 (2C), 122.61, 122.03 (2C), 121.84, 117.91, 26.44; ESI-MS: *m/z* 364.44 (M+H)^+^.

*4-(4-(3-Methoxybenzamido)phenylthio)-N-methylpicolinamide* (**6b**). Orange solid; yield: 35.47%; m.p. 60.1–63.4 °C; ^1^H-NMR (DMSO-*d*_6_): δ 2.77 (d, *J* = 4.8 Hz, 3H), 3.84 (d, *J* = 8.0 Hz, 3H), 7.18 (dd, *J* = 2.4, 8.0 Hz, 1H), 7.25 (dd, *J* = 1.6, 5.2 Hz,1H), 7.45–7.63 (m, 6H), 7.99 (d, *J* = 8.4 Hz, 2H), 8.40 (d, *J* = 5.6 Hz, 1H), 8.69 (d, *J* = 4.4 Hz, 1H), 10.48 (s, 1H); ^13^C-NMR (DMSO*-d*_6_): δ 165.69, 163.78, 159.16, 152.11, 150.14, 148.18, 141.13, 136.09 (2C), 136.05, 129.57, 122.09, 121.57 (2C), 121.38, 119.94, 117.45, 117.39, 113.04, 55.31, 25.93; ESI-MS: *m/z* 394.45 (M+H)^+^.

*4-(4-(4-Methoxybenzamido)phenylthio)-N-methylpicolinamide* (**6c**). Orange solid; yield: 33.20%; m.p. 155.2–158.7 °C; ^1^H-NMR (DMSO-*d*_6_): δ 2.76 (t, *J* = 5.8 Hz, 3H), 3.85 (t, *J* = 9.8 Hz, 3H), 7.09 (d, *J* = 9.2 Hz, 2H), 7.25 (dd, *J* = 2.0, 5.2 Hz, 1H), 7.54 (d, *J* = 1.6 Hz, 1H), 7.61 (d, *J* = 8.4 Hz, 2H), 7.99 (q, *J* = 4.0 Hz, 4H), 8.40 (d, *J* = 4.8 Hz, 1H), 8.75 (d, *J* = 4.8 Hz, 1H), 10.40 (s, 1H); ^13^C-NMR (DMSO*-d*_6_): δ 165.77, 164.29, 162.58, 152.69, 150.65, 148.71, 141.91, 136.55 (2C), 130.24 (2C), 127.14, 122.58, 121.96 (2C), 121.46, 117.88, 114.14 (2C), 55.93, 26.43; ESI-MS: *m/z* 394.43 (M+H)^+^.

*4-(4-(2-Methoxybenzamido)phenylthio)-N-methylpicolinamide* (**6d**). Orange solid; yield: 82.47%; m.p. 122.4–125.1 °C; ^1^H-NMR (DMSO-*d*_6_): δ 2.76 (d, *J* = 4.8 Hz, 3H), 3.91 (s, 3H), 7.08 (t, *J* = 7.6 Hz, 1H), 7.20 (d, *J* = 8.4 Hz, 1H), 7.26 (dd, *J* = 2.0, 5.2 Hz, 1H), 7.51–7.54 (m, 2H), 7.60–7.64 (m, 3H), 7.95 (d, *J* = 8.4 Hz, 2H), 8.41 (d, *J* = 5.6 Hz, 1H), 8.76 (d, *J* = 4.8 Hz, 1H), 10.46 (s, 1H); ^13^C-NMR (DMSO*-d*_6_): δ 165.55, 164.30, 156.94, 152.68, 150.65, 148.70, 141.50, 136.70 (2C), 132.62, 130.07, 125.41, 122.57, 121.61, 121.50 (2C), 120.95, 117.88, 112.44, 56.34, 26.44; ESI-MS: *m/z* 416.14 (M+Na)^+^.

*4-(4-(3,5-Dimethoxybenzamido)phenylthio)-N-methylpicolinamide* (**6e**). Orange solid; yield: 32.13%; m.p. 153.9–157.4 °C; ^1^H-NMR (DMSO-*d*_6_): δ 2.77 (d, *J* = 4.4 Hz, 3H), 3.84 (s, 6H), 6.75 (s, 1H), 7.12 (d, *J* = 2.0 Hz, 2H), 7.26 (dd, *J* = 2.0, 5.2 Hz, 1H), 7.54 (s, 1H), 7.63 (d, *J* = 8.4 Hz, 2H), 8.00 (d, *J* = 8.4 Hz, 2H), 8.41 (d, *J* = 5.2 Hz, 1H), 8.76 (d, *J* = 4.8 Hz, 1H), 10.49 (s, 1H); ^13^C-NMR (DMSO*-d*_6_): δ 165.50, 163.71, 160.36 (2C), 152.27, 150.02, 148.09, 141.08, 136.69, 136.03 (2C), 122.10, 121.64 (2C), 121.39, 117.42, 105.72 (2C), 103.47, 55.48 (2C), 25.93; ESI-MS: *m/z* 424.20 (M+H)^+^.

*4-(4-(2-Chlorobenzamido)phenylthio)-N-methylpicolinamide* (**6f**). Orange solid; yield: 70.04%; m.p. 191.3–193.5 °C; ^1^H-NMR (DMSO-*d*_6_): δ 2.76 (d, *J* = 5.2 Hz, 3H), 7.26 (t, *J* = 2.8 Hz, 1H), 7.46–7.66 (m, 7H), 7.93 (d, *J* = 8.4 Hz, 2H), 8.41 (d, *J* = 5.6 Hz, 1H), 8.76 (d, *J* = 4.8 Hz, 1H), 10.86 (s, 1H); ^13^C-NMR (DMSO*-d*_6_): δ 165.28, 163.80, 152.04, 150.14, 148.22, 140.79, 136.63, 136.25 (2C), 131.26, 129.92, 129.66, 128.96, 127.26, 122.13, 121.66, 120.92 (2C), 117.41, 25.95; ESI-MS: *m/z* 398.40 (M+H)^+^.

*4-(4-(3-Fluorobenzamido)phenylthio)-N-methylpicolinamide* (**6g**). Orange solid; yield: 71.66%; m.p. 187.9–189.0 °C; ^1^H-NMR (DMSO-*d*_6_): δ 2.77 (d, *J* = 4.8 Hz, 3H), 7.27 (d, *J* = 5.2 Hz, 1H), 7.49 (t, *J* = 8.4 Hz, 1H), 7.54 (s, 1H), 7.60–7.65 (m, 3H), 7.83 (q, *J* = 7.7 Hz, 2H), 8.01 (d, *J* = 8.0 Hz, 2H), 8.42 (d, *J* = 5.2 Hz, 1H), 8.76 (d, *J* = 4.4 Hz, 1H), 10.62 (s, 1H); ^13^C-NMR (DMSO*-d*_6_): δ 165.03, 164.26, 163.60, 161.17, 152.57, 150.62, 148.66, 141.40, 137.41, 136.55 (2C), 131.03, 124.46, 122.62, 122.11 (2C), 117.94, 115.22, 114.99, 26.43; ESI-MS: *m/z* 380.17 (M−H)^+^.

*4-(4-(2,4-Dichlorobenzamido)phenylthio)-N-methylpicolinamide* (**6h**). Orange solid; yield: 59.61%; m.p. 227.3–229.7 °C; ^1^H-NMR (DMSO-*d*_6_): δ 2.77 (d, *J* = 4.8 Hz, 3H), 7.26 (dd, *J* = 2.0, 5.2 Hz, 1H), 7.55–7.64 (m, 5H), 7.79 (d, *J* = 1.6 Hz, 1H), 7.90 (d, *J* = 8.4 Hz, 2H), 8.41 (d, *J* = 5.2 Hz, 1H), 8.70 (d, *J* = 4.8 Hz, 1H), 10.84 (s, 1H); ^13^C-NMR (DMSO*-d*_6_): δ 164.35, 163.78, 151.97, 150.15, 148.21, 140.60, 136.27 (2C), 135.41, 135.07, 131.24, 130.38, 129.22, 127.47, 122.15, 121.88, 120.95 (2C), 117.41, 25.94; ESI-MS: *m/z* 432.23 (M+H)^+^.

*4-(4-(2,6-Difluorobenzamido)phenylthio)-N-methylpicolinamide* (**6i**). Orange solid; yield: 64.15%; m.p. 188.2–190.1 °C; ^1^H-NMR (DMSO-*d*_6_): δ 2.78 (d, *J* = 4.8 Hz, 3H), 7.24–7.32 (m, 3H), 7.57 (s, 1H), 7.60–7.66 (m, 3H), 7.89 (d, *J* = 8.4 Hz, 2H), 8.41 (d, *J* = 5.2 Hz, 1H), 8.77 (d, *J* = 4.8 Hz, 1H), 11.15 (s, 1H); ^13^C-NMR (DMSO*-d*_6_): δ 163.69, 159.57, 158.53, 157.97, 152.10, 149.98, 148.17, 140.26, 136.36 (2C), 132.32, 122.23, 122.16, 120.83 (2C), 117.54, 115.11, 112.18, 112.04, 25.95; ESI-MS: *m/z* 422.12 (M+Na)^+^.

*N-Methyl-4-(4-(2,3,4,5-tetrafluorobenzamido)phenylthio)picolinamide* (**6j**). Orange solid; yield: 47.85%; m.p. 183.2–185.7 °C; ^1^H-NMR (DMSO-*d*_6_): δ 2.76 (d, *J* = 5.2 Hz, 3H), 7.27 (dd, *J* = 2.0, 5.2 Hz, 1H), 7.52 (d, *J* = 2.0 Hz, 1H), 7.65 (d, *J* = 8.8 Hz, 2H), 7.83–7.87 (m, 3H), 8.41 (d, *J* = 5.2 Hz, 1H), 8.76 (d, *J* = 4.8 Hz, 1H), 10.95 (s, 1H); ^13^C-NMR (DMSO*-d*_6_): δ 164.02, 160.25, 158.71, 156.87, 152.15, 150.40, 148.45, 140.49, 136.53 (2C), 132.55, 122.58, 122.41, 121.43 (2C), 117.65, 114.93, 112.34, 112.13, 26.16; ESI-MS: *m/z* 458.04 (M+Na)^+^.

*N-Methyl-4-(4-(3-(trifluoromethyl)benzamido)phenylthio)picolinamide* (**6k**). Orange solid; yield: 71.09%; m.p. 168.1–169.5 °C; ^1^H-NMR (DMSO-*d*_6_): δ 2.77 (d, *J* = 4.8 Hz, 3H), 7.26 (dd, *J* = 2.0, 5.2 Hz, 1H), 7.56 (s, 1H), 7.65 (d, *J* = 8.8 Hz, 2H), 7.81 (t, *J* = 7.8 Hz, 1H), 8.00 (d, *J* = 8.8 Hz, 3H), 8.28 (s, 1H), 8.31 (d, *J* = 4.4 Hz, 1H), 8.41 (d, *J* = 5.2 Hz, 1H), 8.70 (d, *J* = 4.4 Hz, 1H), 10.73 (s, 1H); ^13^C-NMR (DMSO*-d*_6_): δ 164.43, 163.72, 152.10, 150.06, 148.12, 140.82, 136.04 (2C), 135.53, 131.89, 129.68, 128.26, 124.80, 124.36, 124.34, 122.12, 121.82, 121.70 (2C), 117.46, 25.91; ESI-MS: *m/z* 432.35 (M+H)^+^.

*N-Methyl-4-(4-(4-(trifluoromethyl)benzamido)phenylthio)picolinamide* (**6l**). Orange solid; yield: 56.73%; m.p. 193.6–194.9 °C; ^1^H-NMR (DMSO-*d*_6_): δ 2.77 (d, *J* = 4.4 Hz, 3H), 7.27 (dd, *J* = 1.6, 5.2 Hz, 1H), 7.55 (s, 1H), 7.65 (d, *J* = 8.8 Hz, 2H), 7.95 (d, *J* = 8.0 Hz, 2H), 8.01 (d, *J* = 8.8 Hz, 2H), 8.18 (d, *J* = 8.0 Hz, 2H), 8.42 (d, *J* = 5.2 Hz, 1H), 8.76 (d, *J* = 4.8 Hz, 1H), 10.78 (s, 1H); ^13^C-NMR (DMSO*-d*_6_): δ 164.79, 163.77, 152.00, 150.16, 148.19, 140.83, 138.47, 136.07 (2C), 128.68 (2C), 125.37, 125.35 (2C), 122.94, 122.12, 121.84, 121.62 (2C), 117.43, 25.91; ESI-MS: *m/z* 432.32 (M+H)^+^.

*N-Methyl-4-(4-(2-(trifluoromethyl)benzamido)phenylthio)picolinamide* (**6m**). Orange solid; yield: 71.22%; m.p. 232.3–233.5 °C; ^1^H-NMR (DMSO-*d*_6_): δ 2.78 (d, *J* = 4.4 Hz, 3H), 7.27 (d, *J* = 4.0 Hz, 1H), 7.56 (s, 1H), 7.64 (d, *J* = 8.4 Hz, 2H), 7.73–7.85 (m, 3H), 7.89 (t, *J* = 6.8 Hz, 3H), 8.42 (d, *J* = 5.2 Hz, 1H), 8.77 (d, *J* = 4.4 Hz, 1H), 10.92 (s, 1H); ^13^C-NMR (DMSO*-d*_6_): δ 165.90, 163.80, 151.99, 150.16, 148.23, 140.76, 136.24 (2C), 135.81, 132.62, 130.21, 128.56, 126.33, 124.63, 122.82, 122.14, 121.79, 120.96 (2C), 117.43, 25.93; ESI-MS: *m/z* 430.00 (M−H)^+^.

*N-Methyl-4-(4-(3-nitrobenzamido)phenylthio)picolinamide* (**6n**). Orange solid; yield: 50.01%; m.p. 200.4–201.1 °C; ^1^H-NMR (DMSO-*d*_6_): δ 2.77 (d, *J* = 4.8 Hz, 3H), 7.26 (dd, *J* = 2.0, 5.2 Hz, 1H), 7.57 (d, *J* = 1.6 Hz, 1H), 7.65 (d, *J* = 8.4 Hz, 2H), 7.86 (t, *J* = 8.0 Hz, 1H), 8.05 (d, *J* = 8.4 Hz, 2H), 8.41 (d, *J* = 5.2 Hz, 1H), 8.46 (dd, *J* = 1.6, 8.4 Hz, 1H), 8.50 (d, *J* = 8.0 Hz, 1H), 8.70 (d, *J* = 5.2 Hz, 1H), 8.82 (s, 1H), 11.02 (s, 1H); ^13^C-NMR (DMSO*-d*_6_): δ 164.27 (2C), 152.46, 150.65, 148.77, 148.18, 141.32, 136.49 (2C), 136.43, 134.89, 130.63, 126.79, 123.21, 122.64, 122.41, 122.36 (2C), 117.96, 26.44; ESI-MS: *m/z* 409.19 (M+H)^+^.

*N-Methyl-4-(4-(4-nitrobenzamido)phenylthio)picolinamide* (**6o**). Orange solid; yield: 78.93%; m.p. 215.9–216.6 °C; ^1^H-NMR (DMSO-*d*_6_): δ 2.77 (d, *J* = 4.4 Hz, 3H), 7.27 (d, *J* = 5.2 Hz, 1H), 7.55 (s, 1H), 7.66 (d, *J* = 8.4 Hz, 2H), 8.03 (d, *J* = 8.4 Hz, 2H), 8.23 (d, *J* = 8.8 Hz, 2H), 8.41 (t, *J* = 7.2 Hz, 3H), 8.76 (d, *J* = 4.4 Hz, 1H), 10.93 (s, 1H); ^13^C-NMR (DMSO*-d*_6_): δ 164.28, 163.83, 151.93, 150.07, 149.15, 148.23, 140.70, 140.13, 135.96 (2C), 129.40 (2C), 123.43 (2C), 122.12, 122.02, 121.77 (2C), 117.45, 25.91; ESI-MS: *m/z* 409.25 (M+H)^+^.

*4-(4-Acetamidophenylthio)-N-methylpicolinamide* (**1**). Orange solid; yield: 74.82%; m.p. 181.7–184.1 °C; ^1^H-NMR (DMSO-*d*_6_): δ 2.10 (s, 3H), 2.76 (d, *J* = 4.8 Hz, 3H), 7.23 (dd, *J* = 2.0, 5.2 Hz, 1H), 7.51 (d, *J* = 1.6 Hz, 1H), 7.56 (d, *J* = 8.8 Hz, 2H), 7.78 (d, *J* = 8.8 Hz, 2H), 8.39 (d, *J* = 5.2 Hz, 1H), 8.74 (d, *J* = 4.8 Hz, 1H), 10.27 (s, 1H); ^13^C-NMR (DMSO*-d*_6_): δ 168.77, 163.77, 152.24, 150.10, 148.14, 141.25, 136.21 (2C), 121.98, 120.44, 120.24 (2C), 117.31, 25.92, 24.09; ESI-MS: *m/z* 324.11 (M+Na)^+^.

*4-(4-(2-Chloroacetamido)phenylthio)-N-methylpicolinamide* (**6p**). Orange solid; yield: 43.93%; m.p. 203.1–205.7 °C; ^1^H-NMR (DMSO-*d*_6_): δ 2.77 (t, *J* = 6.2 Hz, 3H), 4.35 (s, 2H), 7.22 (dd, *J* = 2.0, 4.8 Hz, 1H), 7.53 (d, *J* = 2.0 Hz, 1H), 7.60 (d, *J* = 8.8 Hz, 2H), 7.83 (d, *J* = 8.8 Hz, 2H), 8.40 (d, *J* = 5.2 Hz, 1H), 8.75 (d, *J* = 4.4 Hz, 1H), 10.90 (s, 1H); ^13^C-NMR (DMSO*-d*_6_): δ 165.67, 164.22, 152.63, 150.51, 148.67, 141.10, 136.69 (2C), 122.54, 121.97, 121.15 (2C), 117.97, 44.01, 26.45; ESI-MS: *m/z* 336.34 (M+H)^+^.

*4-(4-(2,2-Dichloroacetamido)phenylthio)-N-methylpicolinamide* (**6q**). Orange solid; yield: 70.01%; m.p. 195.5–196.9 °C; ^1^H-NMR (DMSO-*d*_6_): δ 2.77 (d, *J* = 4.4 Hz, 3H), 6.66 (s, 1H), 7.25 (d, *J* = 4.4 Hz, 1H), 7.54 (s, 1H), 7.65 (d, *J* = 8.4 Hz, 2H), 7.82 (d, *J* = 8.0 Hz, 2H), 8.41 (d, *J* = 5.2 Hz, 1H), 8.75 (d, *J* = 5.2 Hz, 1H), 11.01 (s, 1H); ^13^C-NMR (DMSO*-d*_6_): δ 163.73, 162.07, 151.68, 150.15, 148.26, 139.48, 136.29 (2C), 122.89, 122.21, 121.16 (2C), 117.55, 67.22, 25.94; ESI-MS: *m/z* 368.02 (M−H)^+^.

*N-Methyl-4-(4-(2,2,2-trichloroacetamido)phenylthio)picolinamide* (**6r**). Orange solid; yield: 28.15%; m.p. 197.0–197.8 °C; ^1^H-NMR (DMSO-*d*_6_): δ 2.77 (d, *J* = 4.8 Hz, 3H), 7.27 (dd, *J* = 1.6, 5.2 Hz, 1H), 7.55 (d, *J* = 1.2 Hz, 1H), 7.67 (d, *J* = 8.4 Hz, 2H), 7.90 (d, *J* = 8.4 Hz, 2H), 8.42 (d, *J* = 5.2 Hz, 1H), 8.76 (d, *J* = 4.8 Hz, 1H), 11.15 (s, 1H); ^13^C-NMR (DMSO*-d*_6_): δ 164.28, 160.34, 152.00, 150.63, 148.80, 139.57, 136.46 (2C), 124.38, 123.08 (2C), 122.84, 118.14, 93.31, 26.44; ESI-MS: *m/z* 401.97 (M−H)^+^.

*4-(4-(3-Chloropropanamido)phenylthio)-N-methylpicolinamide* (**6s**). Orange solid; yield: 58.10%; m.p. 145.0–147.2 °C; ^1^H-NMR (DMSO-*d*_6_): δ 2.77 (t, *J* = 6.4 Hz, 3H), 2.88 (t, *J* = 6.2 Hz, 2H), 3.90 (t, *J* = 6.4 Hz, 2H), 7.21 (dd, *J* = 2.0, 5.2 Hz, 1H), 7.53 (d, *J* = 1.2 Hz, 1H), 7.57 (d, *J* = 8.4 Hz, 2H), 7.80 (d, *J* = 8.8 Hz, 2H), 8.39 (d, *J* = 5.6 Hz, 1H), 8.68 (d, *J* = 4.4 Hz, 1H), 10.35 (s, 1H); ^13^C-NMR (DMSO*-d*_6_): δ 168.96, 164.26, 152.64, 150.61, 148.67, 141.37, 136.77 (2C), 122.52, 121.44, 120.90 (2C), 117.86, 60.21, 26.42, 14.52; ESI-MS: *m/z* 372.19 (M+Na)^+^.

*4-(4-(4-Chlorobutanamido)phenylthio)-N-methylpicolinamide* (**6t**). Orange solid; yield: 76.45%; m.p. 106.4–108.9 °C; ^1^H-NMR (DMSO-*d*_6_): δ 2.03–2.10 (m, 2H), 2.54 (q, *J* = 7.6 Hz, 2H), 2.76 (d, *J* = 5.2 Hz, 3H), 3.72 (t, *J* = 6.4 Hz, 2H), 7.23 (d, *J* = 4.0 Hz, 1H), 7.51 (s, 1H), 7.56 (d, *J* = 8.8 Hz, 2H), 7.80 (d, *J* = 8.4 Hz, 2H), 8.40 (d, *J* = 5.2 Hz, 1H), 8.75 (d, *J* = 4.8 Hz, 1H), 10.33 (s, 1H); ^13^C-NMR (DMSO*-d*_6_): δ 170.71, 163.53, 152.83, 149.67, 147.84, 141.22, 136.62, 136.20 (2C), 122.01, 120.37 (2C), 117.43, 44.94, 33.47, 27.76, 25.95; ESI-MS: *m/z* 398.05 (M+Cl)^−^.

*N-Methyl-4-(4-propionamidophenylthio)picolinamide* (**6u**). Orange solid; yield: 53.49%; m.p. 184.6–187.0 °C; ^1^H-NMR (DMSO-*d*_6_): δ 1.11 (t, *J* = 7.6 Hz, 3H), 2.38 (q, *J* = 7.6 Hz, 2H), 2.76 (d, *J* = 4.8 Hz, 3H), 7.23 (dd, *J* = 1.6, 5.2 Hz, 1H), 7.50 (s, 1H), 7.56 (d, *J* = 8.4 Hz, 2H), 7.80 (d, *J* = 8.4 Hz, 2H), 8.39 (d, *J* = 5.2 Hz, 1H), 8.75 (d, *J* = 4.4 Hz, 1H), 10.20 (s, 1H); ^13^C-NMR (DMSO*-d*_6_): δ 172.73, 164.05, 152.57, 150.35, 148.38, 141.61, 136.50 (2C), 122.22, 120.53 (2C), 117.55, 29.90, 26.21, 26.18, 9.77; ESI-MS: *m/z* 314.10 (M−H)^+^.

*4-(4-Butyramidophenylthio)-N-methylpicolinamide* (**6v**). Orange solid; yield: 84.96%; m.p. 144.8–147.0 °C; ^1^H-NMR (DMSO-*d*_6_): δ 0.94 (t, *J* = 7.4 Hz, 3H), 1.59–1.67 (m, 2H), 2.34 (t, *J* = 7.4 Hz, 2H), 2.77 (d, *J* = 4.8 Hz, 3H), 7.23 (d, *J* = 4.8 Hz, 1H), 7.51 (s, 1H), 7.56 (d, *J* = 8.0 Hz, 2H), 7.80 (d, *J* = 8.4 Hz, 2H), 8.39 (d, *J* = 5.2 Hz, 1H), 8.74 (d, *J* = 4.8 Hz, 1H), 10.21 (s, 1H); ^13^C-NMR (DMSO*-d*_6_): δ 171.61, 163.77, 152.26, 150.10, 148.13, 141.27, 136.20(2C), 121.97, 120.37(2C), 120.30, 117.31, 38.40, 25.91, 18.45, 13.57; ESI-MS: *m/z* 328.12 (M−H)^+^.

*N-Methyl-4-(4-pivalamidophenylthio)picolinamide* (**6w**). Orange solid; yield: 51.57%; m.p. 108.5–110.5 °C; ^1^H-NMR (DMSO-*d*_6_): δ 1.26 (s, 9H), 2.76 (d, *J* = 4.8 Hz, 3H), 7.24 (d, *J* = 5.2 Hz, 1H), 7.51 (s, 1H), 7.56 (d, *J* = 8.4 Hz, 2H), 7.89 (d, *J* = 8.4 Hz, 2H), 8.40 (d, *J* = 5.2 Hz, 1H), 8.75 (d, *J* = 4.8 Hz, 1H), 9.50 (s, 1H); ^13^C-NMR (DMSO*-d*_6_): δ 177.35, 164.28, 152.76, 150.62, 148.64, 141.92, 136.42 (2C), 122.53, 121.82 (2C), 121.12, 117.83, 27.55 (4C), 26.42; ESI-MS: *m/z* 366.23 (M+Na)^+^.

### 3.5. Cell Culture

Cell lines including HepG2, A375 and U87 were maintained in Dulbecco’s modified Eagle medium (DMEM) containing 10% fetal bovine serum (FBS), penicillin (100 U/mL) and streptomycin (10 mg/L). Cell lines including HCT116, MCF-7, SPC-A1, A549 and SW480, were maintained in RPMI 1640 containing 10% fetal bovine serum (FBS), penicillin (100 U/mL) and streptomycin (10 mg/L). Cells were grown in a 5% CO_2_ incubator at 37 °C.

### 3.6. Cell Proliferation Assay (MTT Assay)

Cells (3 × 10^3^/well) were seeded in 96-well plates and cultured for 24 h, followed by treatment with the compounds for 48 h. Ten microliters of 10 mg/mL MTT was added per well and incubated for another 2.5 h at 37 °C. Then the supernatant fluid was removed and 150 μL/well DMSO was added for 15–20 min. The absorbance (OD) of each well was measured at 570 nm using an ELISA reader (Thermo). The effect of compounds on tumor cells viability was expressed by IC_50_ of each cell line.

### 3.7. Kinase Inhibitory Assay

*In vitro* kinase inhibitory assays were performed against recombinant human Aurora-B kinase at the Km of ATP (15 μM) and at a fixed concentration of 10 μM of test compound. Each assay was repeated twice. All the inhibitory assays against Aurora-B were carried out through kinase profiling services provided by Millipore (America), in which radiometric protein kinase assays were used.

## 4. Conclusions

In conclusion, a series of novel *N*-methylpicolinamide-4-thiol derivatives has been synthesized and evaluated on human cancer cell lines. Among them, compound **6p** was found to be the most potent, displaying broad-spectrum *in vitro* antiproliferative activities. The results of the MTT assay showed that compound **6p** had significant cytotoxicity against liver cancer cell line HepG2, colon cancer cell lines HCT-116 and SW480, lung cancer cell line SPC-A1 and melanotic cancer cell line A375 with IC_50_ values <10 μM. All these antiproliferative activities were better than those of the reference compound sorafenib. The advanced kinase inhibitory assays, which were performed on six kinases at a concentration of 10 μM, indicated that **6p** could selectively inhibit Aurora-B kinase. A molecular docking study showed the stable interactions of **6p** with the Aurora-B kinase, which rationalized the obtained biological results. Our ongoing work aimed at researching the advanced mechanism of action and explore the efﬁcacy of compound **6p** in a range of *in* *vivo* models, will be the subjects of future reports.
